# Supplementary material for: Impact of Plasmodiophora brassicae on Canola Root and Rhizosphere Microbiomes and Its Implications for Clubroot Biocontrol
Source: Pathogens. 2025 Sep 9;14(9):904. doi: 10.3390/pathogens14090904 (PMC12472839; doi:10.3390/pathogens14090904)
Supplement: Supplementary file 1 [file pathogens-14-00904-s001.zip › pathogens-3808537-supplementary.pdf]

Supplementary Tables

Table S1. Total number of sequences and average reads per sample of bacteria and fungi associated with the seeds, soil, rhizosphere, and roots of clubroot-susceptible and clubroot-resistant canola lines.

	Bacteria	
	Total number of reads	Average reads/sample
Seeds	2,287	127
Soil mixture	193,113	193,113
Rhizosphere	6,723,113	62,251
Root	1,917,359	17,753
	Fungi	
	Total number of reads	Average reads/sample
Seeds	575	34
Soil mixture	81,677	27,225
Rhizosphere	4,197,491	38,865
Root	1,248,231	11,557

Supplementary Figures

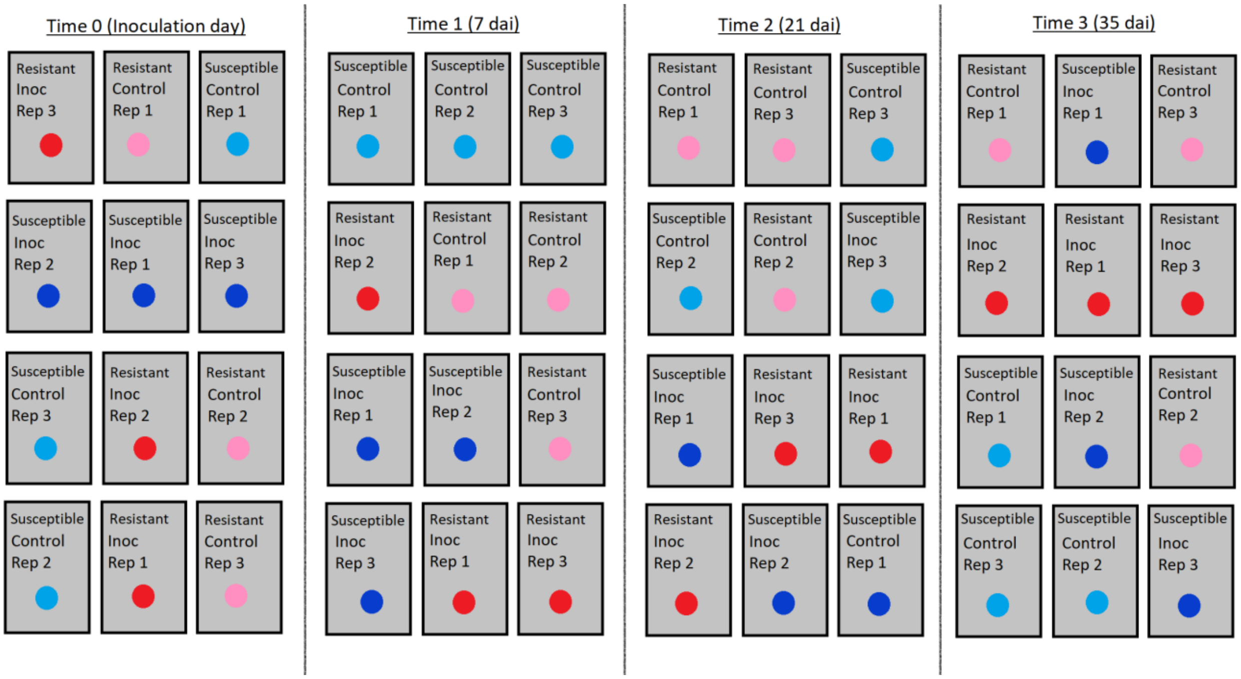

Figure S1. Schematic representation of the experimental treatments and replicates in the greenhouse study assessing the effect of *Plasmodiophora brassicae* (pathotype 3A) inoculation on the fungal and bacterial microbiomes of clubroot-susceptible and clubroot-resistant canola lines. Samples were collected at 7, 21, and 35 days after inoculation (dai). Biological replicates were arranged in a completely randomized design and rotated on each harvest date.

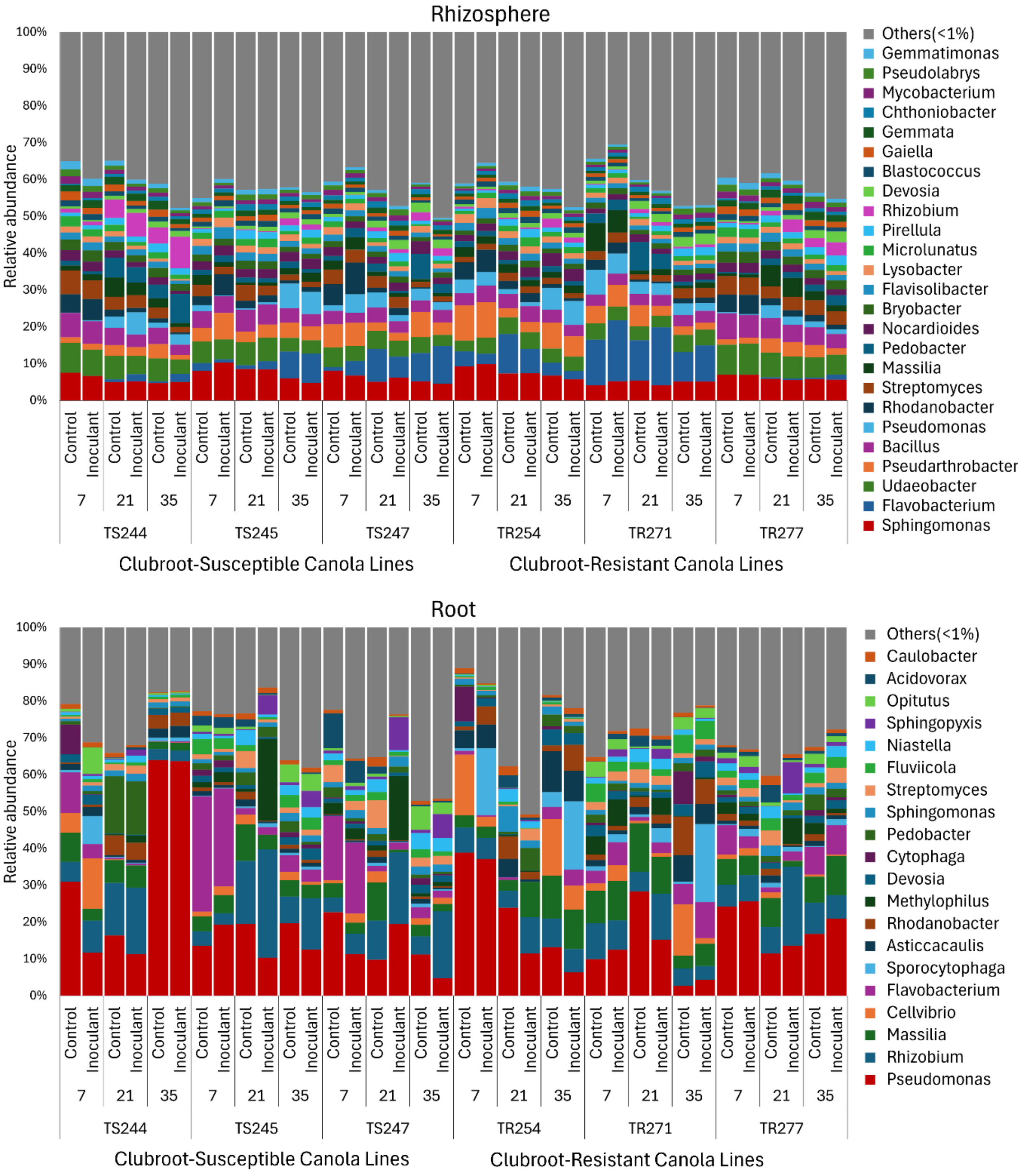

Figure S2. Relative abundance of bacterial genera associated with rhizosphere and root samples (N=3) of clubroot-susceptible and clubroot-resistant canola lines. Plants were inoculated with *Plasmodiophora brassicae* (pathotype 3A), and non-inoculated plants served as controls. Samples were collected at 7, 21 and 35 dai.

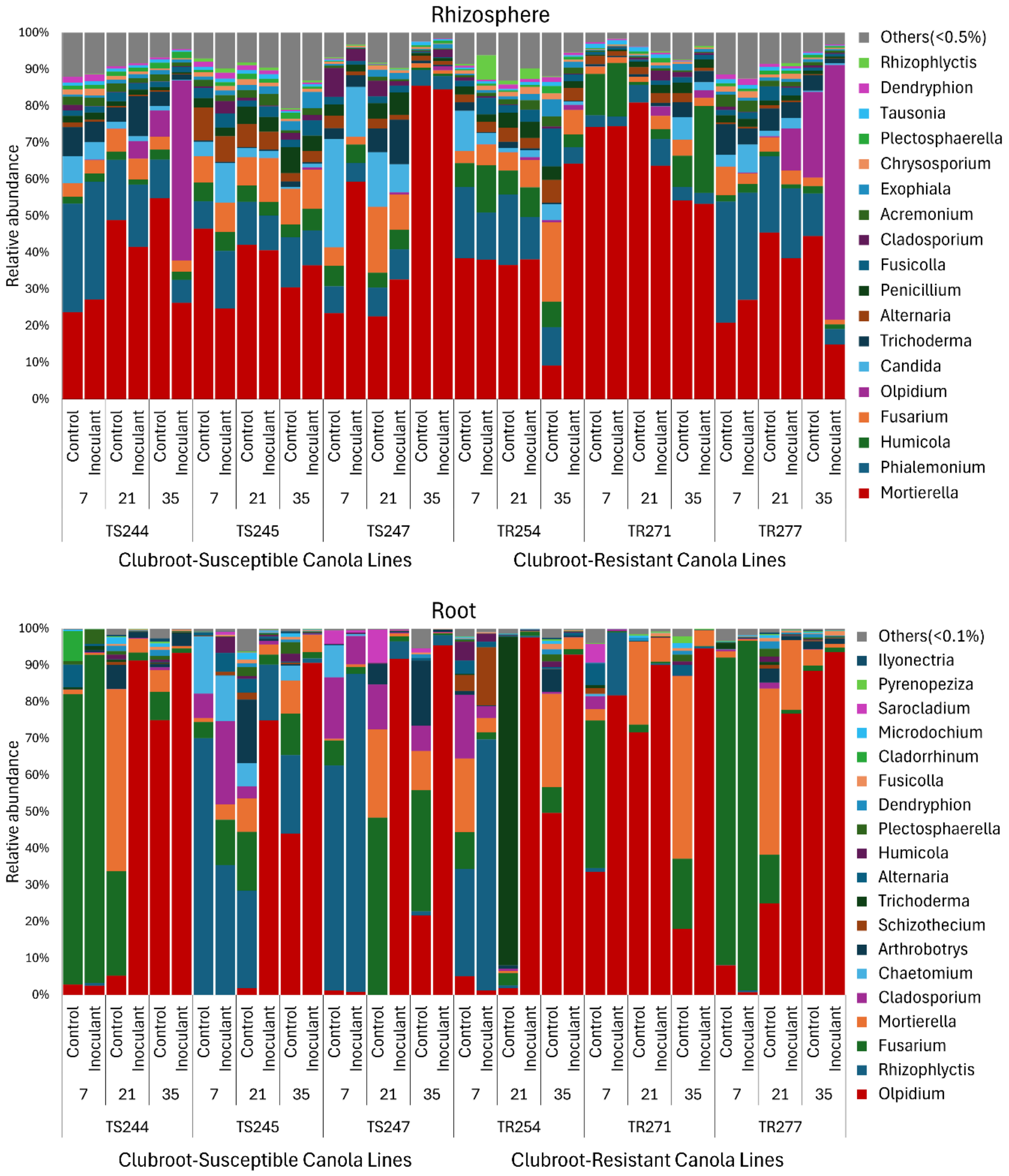

Figure S3. Relative abundance of fungal genera associated with rhizosphere and root samples (N=3) of clubroot-susceptible and clubroot-resistant canola lines. Plants were inoculated with *P. brassicae* (pathotype 3A), and non-inoculated canola plants served as controls. Samples were collected at 7, 21 and 35 dai.
